# Sequential Imaging Characteristics and Potential Role of F18 Fluorodeoxyglucose Positron Emission Tomography/CT in the Evaluation of Treatment Response in Cases of Spinal Tuberculosis Without Neurological Involvement: Results From a Pilot Study

**DOI:** 10.7759/cureus.26065

**Published:** 2022-06-18

**Authors:** Ankit Rai, Anshul Dahuja, Ranjeet Choudhary, Amit Sharma, Shantilal Sankhla

**Affiliations:** 1 Orthopedics, All India Institute of Medical Sciences, Jodhpur, IND; 2 Orthopedics, Guru Gobind Singh Medical College, Faridkot, IND

**Keywords:** fdg pet/ct, non-invasive, anti-tubercular therapy (att), non-surgical treatment, spinal tuberculosis

## Abstract

Purpose

In this non-randomized study, we prospectively studied the sequential imaging properties of fluorodeoxyglucose (FDG) positron emission tomography/computed tomography (PET/CT) and evaluated the role of FDG PET as a non-invasive imaging modality for identifying non-responders during anti-tubercular treatment (ATT) of spinal tuberculosis (TB).

Methods

Before starting anti-tubercular treatment, 25 patients with clinically and radiological suspected; pathologically confirmed spinal TB had a pretreatment contrast-enhanced whole-body FDG PET scan, followed by scans at six, 12, and 18 months. The maximum standardized uptake value (SUVmax) was computed, and the mean change in SUVmax was compared. The mean change in SUVmax was correlated with the clinicoradiological improvement.

Result

In cases of spinal tuberculosis, the FDG PET scan can help identify extra-spinal and non-contagious involvement. In our 25 cases of spinal TB, the baseline peak SUVmax of lesions ranged from 6.3 to 28.5 (mean 14.8). Despite treatment, the condition progressed in two patients, and they had neurological deficits; in both cases, the SUVmax levels increased. The fall in SUVmax during the treatment course was statistically significant (p-value <0.05) and correlated well with the clinical improvement.

Conclusion

The inflammatory cells show increased uptake of F18 FDG, so uptake of radioactive tracer localizes and quantifies the disease activity; thus, FDG PET/CT holds a promising role as a sensitive non-invasive modality for the detection, staging, assessing disease activity, and monitoring therapy and deciding end point treatment in spinal TB.

## Introduction

Spinal tuberculosis (TB) is one of mankind's oldest diseases, with evidence dating back to Egyptian mummies [1]. Spinal TB is believed to cause 15% of extrapulmonary tuberculosis cases and 2% of all tuberculosis cases. It is a substantial burden on global health care due to its high prevalence and lifelong disability [2].

Because of the paucibacillary nature of the disease, the ambiguous clinical picture, subtle radiological abnormalities, and minor and variable changes in biochemical markers, diagnosing spinal tuberculosis is challenging. This leads to misdiagnosis and a delay in diagnosis, both of which contribute to the disease's morbidity [3].

Evaluating the response to anti-tubercular treatment (ATT) and deciding on the completion of treatment is still challenging because serological and radiological markers lag behind the clinical improvement. It is also possible that different investigation modalities can't tell the difference between active infection and latent illness; compromised host immune response also makes follow-up of treatment difficult [4,5].

Fluorodeoxyglucose positron emission tomography/computed tomography (FDG PET/CT) is based on the quantification of metabolic activity in the inflammatory cells, so it plays a significant role in the diagnosis of infectious and inflammatory diseases like tuberculosis. Fluorodeoxyglucose (FDG) gets accumulated in the inflammatory cells and remains non-metabolized; the resultant radioactivity corresponds to the metabolic activity of the inflammatory cells at that site, which is measured as the maximum standardized uptake value (SUVmax). Because of these features, FDG is a suitable radiotracer for monitoring granulomatous inflammatory lesions [6-8].

## Materials and methods

Study design

The current study was an observational non-randomized prospective study that was conducted on patients who presented with signs and symptoms of spinal tuberculosis in the outpatient department (OPD) at a tertiary care center. Clearance was obtained from the Faculty of Medical Sciences, Guru Gobind Singh Medical College and Hospital, Faridkot.

Inclusion and exclusion criteria

Patients over the age of 18 who were clinicoradiologically suspected and pathologically confirmed to have spinal TB and were not on ATT were included in the study. Patients who were below the age of 18, who had uncontrolled diabetes, pregnancy, and known contemporaneous malignancy, immune-compromised state, or concomitant chronic illness, a neurological deficit, those already on ATT, and patients who did not give consent to participate in the study were excluded.

Data collection

After admission, patients had routine blood tests such as complete blood counts and erythrocyte sedimentation rate (ESR). All patients had a full-body FDG PET/CT scan (Siemens Biograph 16 PET/CT Tech Specs; Siemens Healthineers, Erlangen, Germany) before commencing ATT at six, 12, and 18 months. A standard protocol was followed for the PET/CT scan. Following an intravenous (IV) injection of F18 FDG, FDG-PET/CT was conducted 60 minutes later. A PET/CT scanner was used to obtain images from head to thighs. Slices were reformatted into axial, coronal, and sagittal views after images were reconstructed using a three-dimensional virtual unenhanced technique. The patient's weight was taken into account when calculating the maximal standardized uptake value (SUV). In a predefined format, all of the patients' demographic data, clinical and treatment histories, general and systemic examination findings, laboratory parameters, and relevant radiological imaging findings were systematically recorded. The Oswestry disability index was calculated for all patients.

All of the patients in the study had a CT-guided biopsy to confirm the diagnosis of tuberculosis pathologically. Presumptive proof for active tuberculosis was the presence of acid-fast bacilli on Ziehl-Neelsen (ZN) stain or the appearance of granulomatous inflammation with necrosis/caseation. In a few patient samples, culture was done and reported six weeks later. In 14 of the 20 cultures, *Mycobacterium tuberculous* was identified. All the patients who participated in the study were given a four-drug regimen (H isoniazid, R rifampicin, Z pyrazinamide, E ethambutol) for two months, and a two-drug regimen (HR) for 16 months of ATT as per the Revised National TB Control Programme (RNTCP) guidelines.

Statistical analysis

Data was entered and analyzed in SPSS version 20 (IBM Inc., Armonk, New York). Patients were divided into groups according to the length of follow-up. Descriptive statistics were used to summarize the clinical and demographic profiles of all the patients. Repeated measures analysis of variance test was used to compare the mean change in SUVmax level from baseline to 6, 12, 18, 6 to 12, 6 to 18 and 12 to 18 months. A p-value of <0.05 was considered to be statistically significant.

## Results

**Figure 1:**
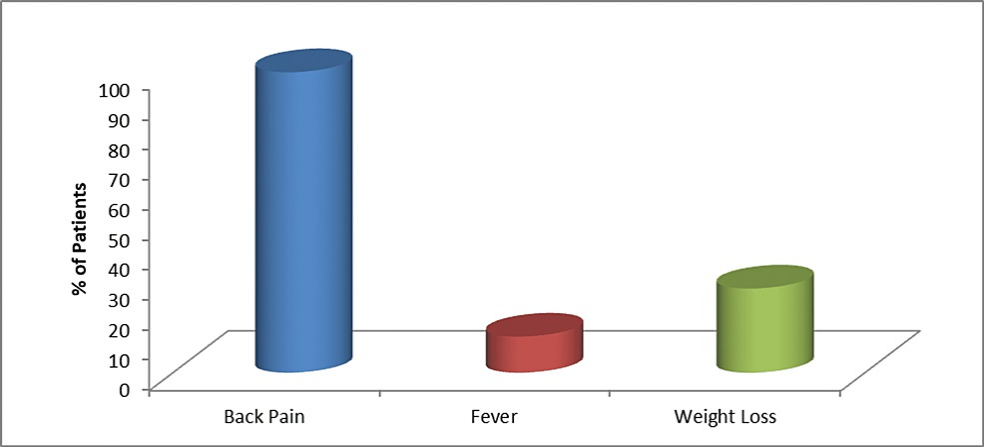
Clinical symptoms at presentation

The study included 25 patients with 11 females (44%) and 14 males (56%). The mean age of patients was 48.7 years. The majority of patients (64%) had lumbar involvement; whereas one patient had non-contagious multilevel involvement. Sixty percent of patients had extra spinal involvement. Lymph nodes were involved in eight cases, uterine in one case, intestinal tuberculosis in one case, and psoas abscess and paraspinal muscle involvement were seen in five cases. Back pain was the most common presenting complaint (100%), followed by weight loss (28%) and fever (12%) (Figure 1).

At presentation, most, but not all, patients had an increase in ESR, with baseline levels ranging from 30-130 (mean 76, 2SD 24.4). The mean SUVmax value before commencing the ATT in our study was 14.87 (range 6.3-28.5). The mean change in SUVmax on sequential FDG PET/CT at various time points from pretreatment to six, 12, and 18 months of ATT were calculated and found to be highly significant (p<0.05) (Table 1). The mean percentage change in the SUVmax from the baseline to the 18 months of treatment was 84.61 and was found to be statistically significant (p<0.05). The mean change in SUVmax correlated well with the improvement in the clinical status of the patient (p=0.001).

**Table 1:** Fall in mean SUVmax during antitubercular treatment. SUVmax - maximum standardized uptake value

SUVmax	N	Range	Minimum	Maximum	Mean	SD
Baseline	25	22.20	6.30	28.50	14.8760	5.70302
6 months	22	11.40	3.80	15.20	9.0773	3.42983
12 months	20	4.10	2.30	6.40	4.3200	1.25974
18 months	19	1.70	1.40	3.10	2.2895	0.57821

17 of the 25 patients were mobile and capable of independent care after six months of ATT. Two of the patients developed neurological problems, which were treated surgically. The tissue culture taken at the time of surgery was positive for multidrug-resistant tuberculosis (MDR TB) in one patient. In these two patients, a rise in SUVmax was noted despite treatment (SUVmax>10). At 12 months of the treatment, two patients were lost to follow-up, and one was lost at 18 months. After 12 months of ATT, all 21 patients reported an improvement in their quality of life and ability to self-care. More than half of the patients were returned to their pre-disease level after 18 months of treatment.

## Discussion

Spinal TB is the most prevalent form of extrapulmonary TB, with permanent lifelong sequelae that necessitate prompt diagnosis and treatment to avoid deformity and neurological deficits [9]. The medical management of spinal TB proves to be very effective, but the long duration of treatment with variable compliance and lack of tools to adequately monitor the treatment response and bacteriological activity resulted in the emergence of MDR and extensively drug-resistant (XDR) TB. FDG PET has emerged as an important tool in the management of cancer patients. F18 FDG accumulates in the inflammatory cells - the intensity of uptake corresponds to inflammatory activity; thus, it not only localizes the lesion but also quantifies the inflammatory activity [10].

In the present study, we saw high FDG uptake localized to the areas of the spine involved in tuberculosis. The baseline SUVmax varies from 6.30 to 28.50 (mean 14.87, 2SD 5.70), which is extremely variable and makes establishing a cut-off value for defining inflammation using semi-quantitative FDG uptake study problematic; similar observations were made in previous studies [11, 12].

We've encountered multilevel noncontiguous spinal tuberculosis in 4% of cases, which is consistent with the majority of studies [13]. Multilevel non-contagious involvement is generally missed with routine diagnostic modalities, but FDG PET/CT identifies not only the multilevel spinal involvement but also other sites of occult pulmonary or extrapulmonary tuberculosis [14]. Raised ESR was seen in most but not all cases of the study group. It has been reported in the literature that patients may have normal ESR in up to 10% of the cases, which explains similar findings in our study [9].

**Figure 2:**
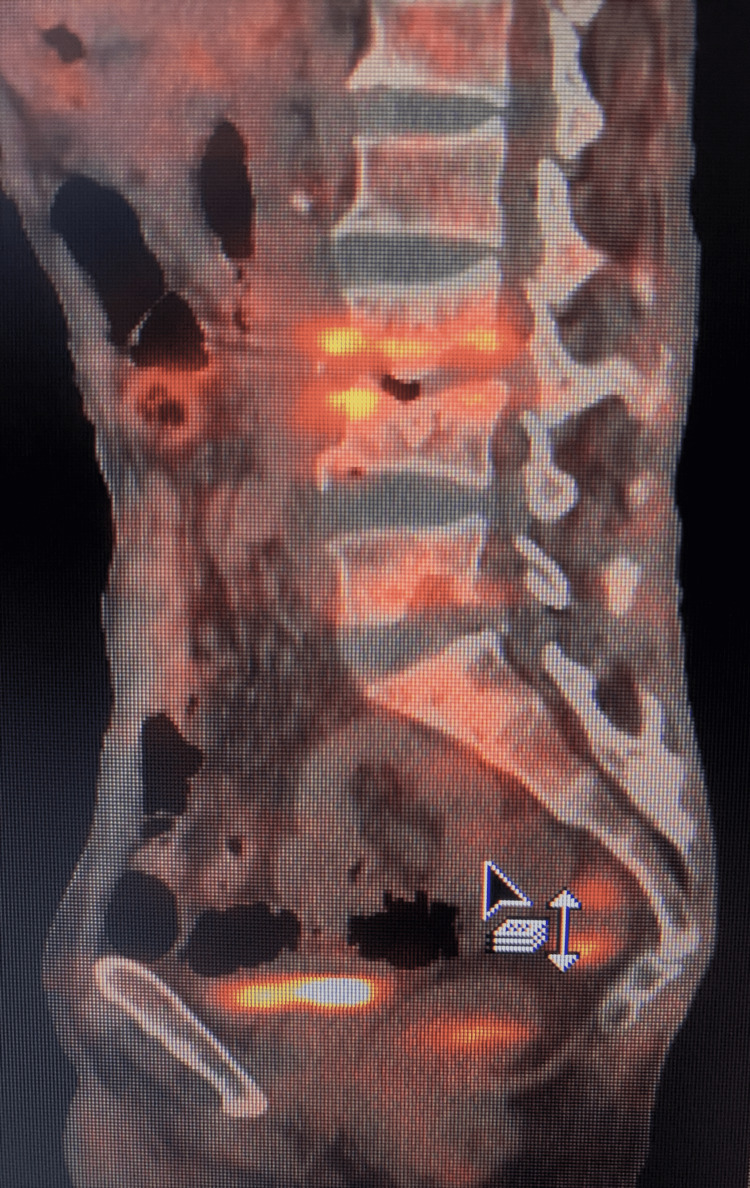
FDG PET/CT of the spine FDG PET/CT showing degenerative changes of the spine along with the collapse of L3 vertebra, end plate changes with the erosion of superior end plate of L4 and inferior end plate of L3 vertebra Increased FDG uptake was seen in L3 - L4 intervertebral disc (SUVmax = 6.3) suggestive of residual disease. FDG - fluorodeoxyglucose, PET - positron emission tomography, SUVmax - maximum standardized uptake value

**Figure 3:**
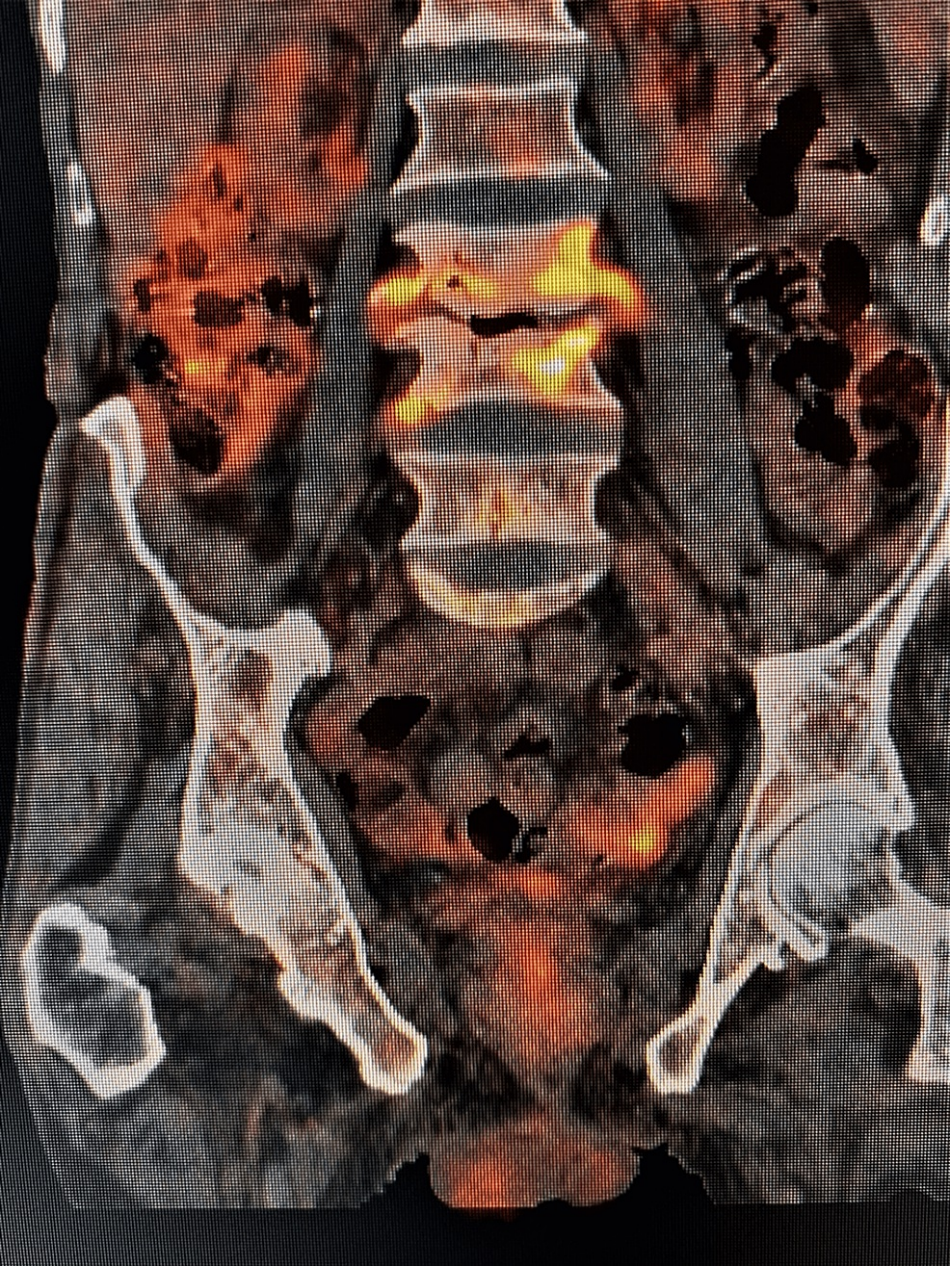
FDG PET/CT of the same patient as above after six months showing a decrease in FDG uptake in L3 - L4 intervertebral disc (SUVmax = 4.1) FDG - fluorodeoxyglucose, PET - positron emission tomography, SUVmax - maximum standardized uptake value

We have observed in the current study, that as treatment progresses, there is a significant fall in mean SUVmax (Figure 2, 3), which corresponds to the decrease in the inflammatory activity. This is in agreement with the observations made by Zinn et al. and Dureja et al. [9, 15]. The change in SUVmax values correlates well with clinical improvement and the ability to perform the daily routine of the patients. This emphasizes that change in SUVmax values can be used as a biomarker for early therapeutic response evaluation. In a few studies, only one month of ATT has shown a significant fall in SUVmax values, which further consolidates our results [16].

Two patients in the present study did not respond to ATT and developed neurological deficit within six months of the start of treatment and needed surgical intervention, where debridement was done, and a tissue sample was taken for culture and histopathological examination. The SUVmax values showed a rising trend in both these cases. FDG PET surpasses other radiological, pathological, and biochemical markers, which are either nonspecific, time-consuming, or dependent on the bacterial isolate, by allowing us to identify non-responders and cases with MDR TB and XDR TB, allowing prompt measures to be taken and treatment to be modified based on response. This helps to prevent the development of drug resistance as well as decrease the real-time assessment of new ATT drugs [17].

Because there is no consensus on the treatment's end-point, which ranges between six, nine, 12, and 18 months, a longer follow-up is required to determine the recurrence rate among patients who had a fall in metabolic activity on FDG PET. Although more research is needed, FDG PET may also have the potential as a non-invasive modality for determining the end point of treatment in extra pulmonary tuberculosis, which is already a point of conflict [18, 19]. Studies with a larger sample size and longer follow-up are needed to emphasize the routine use of FDG PET/CT in evaluating the treatment response of ATT in spinal TB and determining the treatment end-point while keeping the cost benefit in mind.

## Conclusions

FDG PET/CT acts as a sensitive modality that picks out the metabolic activity of inflammatory cells at the pathological site. It identifies the occult sites of involvement either in the spine or elsewhere in the body that are usually missed with other modalities. Serial quantification of metabolic activity on FDG PET/CT allows it to be useful as a non-invasive measure to keep track of treatment response to ATT in spinal TB and early identification of non-responders. Thus, it reduces the delay in the diagnosis of MDR and XDR tuberculosis. Clinical improvement with insignificant soft tissue and bone uptake on the FDG PET/CT may indicate treatment completion, but this role will require a long-term and comprehensive follow-up.
